# Rosalinda Dimapilis-Baldoz: balancing domestic and international needs for health workers

**DOI:** 10.2471/BLT.19.030519

**Published:** 2019-05-01

## Abstract

Rosalinda Dimapilis-Baldoz talks to Fiona Fleck about the health worker migration policy of the Philippines and discusses the challenges faced by the *WHO Global code of practice on the international recruitment of health personnel*.

Q: How did you become involved in labour issues?

A: I worked in labour arbitration as a lawyer in government for more than 20 years before taking the position of Undersecretary for Labour Relations and Management at the Department of Labour and Employment in the late 1990s.

Q: Was that where you started working on the rights of overseas workers?

*A: *Yes, one of my roles was heading up the Philippine Overseas Employment Administration (POEA) and I worked on a number of labour law cases, which involved recruitment violations, contract term violations, contract substitution and misrepresentation, to illegal recruitment and human trafficking.

Q: What do you consider your greatest achievement from that time?

A: During my term, the POEA achieved the greatest volume of refunds to workers for excessive placement fees, along with other monetary awards. So that was one achievement. On the regulatory side, I worked with the Domestic Work Committee of the International Labour Organization (ILO), which led to the adoption of the ILO convention protecting the rights of overseas domestic workers, a convention the Philippines ratified and implemented. We became the first country to negotiate and conclude a bilateral agreement on the protection of female workers with Saudi Arabia in 2013, aligning the rights of domestic workers with those embodied in the ILO convention. Since then we have concluded and signed similar agreements with many other countries on health workers, particularly on nurses and care givers.

Q: The Philippines has become a major source country for health workers. Does this create shortages of health workers at home?

A: To date, we are not suffering any shortages in general nurses, but we do lack specialists and nurses with long experience.

Q: Does the government intend to restrict health worker migration?

A: So far, there has been no call for a ban on health workers seeking overseas employment on the grounds of what we call a "mission-critical skill shortage”, something we have implemented in the past for aircraft mechanics and pilots.

“We are a supplier of health workers to the world, but we also monitor and meet our domestic requirements.”

Q: So, is worker migration something that the Philippines encourages or discourages?

A: We encourage our health workers to weigh the pros and cons before going to work abroad, especially the high social cost of leaving their families and children behind. Broadly speaking, the Philippines considers the health workforce to be a productive investment. Also, because many health workers eventually return to the Philippines, there is a transfer of knowledge over the long term, which is beneficial to the country. But we also want to support other countries and the *Philippine health agenda 2016-2022* includes strategies for harnessing the power of human resources for health development to attain the goal of universal health care and to alleviate the shortage in the global market.

Q: So, addressing the shortage of health workers in the global market is written into national health policy?

A: That’s right. As administrator of the POEA for almost eight years and Secretary of Labour and Employment for six years, where I chaired both the POEA Governing Board and the Overseas Workers Welfare Administration Board of Trustees, I was responsible for opening the foreign markets where we send our nurses and other health workers. At the POEA, we issue policies that regulate and manage the flow of our migrant workers in more than 200 countries, including our health workers. So, we are a supplier of health workers to the world, but we also monitor and meet our domestic requirements.

Q: You worked on the WHO Global Code of Practice on the International Recruitment of Health Personnel. Nine years later, how would you characterize it?

A: I see it as an important guide on principles and measures that destination countries can take to ensure ethical recruitment of health professionals and for origin countries to better manage international migration of health professionals and ensure the sustainability of the supply of personnel for their domestic health systems.

Q: To what extent has the code informed your country’s policies?

A: For the Philippines, the code is an important reference in terms of strategic responses to health personnel development challenges.

Q: The code was adopted by WHO Member States in 2010, but large numbers of health workers are still being actively recruited from countries with critical shortages. How successfully does the code address these problems?

A: It is a very new code and it is voluntary in character. So, it is quite a challenge to achieve full compliance. But compliance is not the only issue. I think more could be done in terms of information management. Getting the balance of supply and demand right requires a greater focus on data collection and use. A study presenting the first round of monitoring on the implementation of the code which was published in this journal (*Monitoring the implementation of the WHO Global code of practice on the international recruitment of health personnel, 2013*) highlighted the wide variety in countries’ capacity to gather data and conduct research on matters relating to health personnel migration. The study found that the lack of coordinated and comprehensive data on health personnel migration was a major obstacle to implementation of the code. These different issues need to be addressed if countries are going to be able to formulate migration policies for domestic requirements and for overseas markets.

Q: What is the Philippines doing in this area?

A: The Department of Health plans to develop an integrated system for the collection of data related to human resources for health from the different government agencies involved in human resources for health development, employment, including worker migration, and reintegration of those who work overseas until retirement in the health workforce. It is seeking WHO assistance in the development of this system.

Q: Can you cite any notable success stories in terms of the international ethical recruitment of health workers that are informed by the code?

A: The Germany-Philippines Bilateral Labour Agreement is one example. It’s a government-to-government arrangement that was signed in 2014 as part of the Triple Win project.

“I believe that addressing the abuses of international ethical recruitment standards is the joint responsibility of both employers and workers, placement agencies, and governments of sending and receiving countries.”

Q: Can you tell me more about that?

A: The Triple Win project is designed to help address the workforce needs of Germany’s health-care sector while at the same time providing job opportunities for the participating countries, which are the Philippines, Serbia, Bosnia and Herzegovina, and Tunisia. Germany recruits qualified nurses for nursing and elderly care facilities.

Q: So Germany recruits health workers in the Philippines?

A: No, the POEA handles the recruitment of the applicants, ensuring that they get the necessary training and that the employers they are going to are accredited. Nurses undergo preparatory German language training in the Philippines prior to their departure for employment in Germany and further language training at the jobsite, along with on-the-job training or immersion in the German health-care system that prepares them to qualify to be licensed nurses in Germany. Once they have finished additional training, they are deployed and are treated the same as German nationals in terms of wages and conditions of employment.

Q: Which aspects of the agreement make it a success story?

A: The agreement sets the standards on legal and ethical recruitment of health professionals consistent with the laws and practices of both countries and is aligned with the principles advocated by the WHO *Code of Practice on International Recruitment of Health Professionals* and the UN Commission on Health Employment and Economic Growth.

Q: Can you give a concrete example of that?

A: For example, under the agreement, Germany renounces the recruitment of nurses from countries that have a shortage of health-care staff. Instead, it taps into the potential of labour markets of countries with overcapacities in the health-care sector, like the Philippines. 

Q: In the meantime, violations and abuses of international ethical recruitment standards continue. What would you say to governments that can stop this?

A: I believe that addressing the abuses of international ethical recruitment standards is the joint responsibility of both employers and workers, placement agencies, and governments of sending and receiving countries. So it needs the support and cooperation of all stakeholders and the global migration community. This is why the UN Commission advocates the promotion of the ILO decent work standards and the WHO Code of Practice, which I, as Commissioner, fully support. To pursue this, the Commission has called on ILO, the Organisation for Economic Co-operation and Development, and WHO to work with relevant partners to establish an international platform on health worker mobility with a view to advancing dialogue, knowledge and cooperation in this space. The Philippines fully supports this recommendation.

